# Optical Measurement of Molar Absorption Coefficient of HbA1c: Comparison of Theoretical and Experimental Results

**DOI:** 10.3390/s22218179

**Published:** 2022-10-25

**Authors:** Shifat Hossain, Shama Satter, Tae-Ho Kwon, Ki-Doo Kim

**Affiliations:** 1 Department of Electrical and Computer Engineering, University of Central Florida, Orlando, FL 32816, USA; 2 Department of Electronics Engineering, Kookmin University, Seoul 02707, Korea

**Keywords:** glycated hemoglobin, HbA1c, diabetes, molar absorption coefficient, optical measurement, noninvasive measurement

## Abstract

Diabetes can cause dangerous complications if not diagnosed in a timely manner. The World Health Organization accepts glycated hemoglobin (HbA1c) as a measure of diagnosing diabetes as it provides significantly more information on the glycemic behavior from a single blood sample than the fasting blood sugar reading. The molar absorption coefficient of HbA1c is needed to quantify the amount of HbA1c present in a blood sample. In this study, we measured the molar absorption coefficient of HbA1c in the range of 450 nm to 700 nm using optical methods experimentally. We observed that the characteristic peaks of the molar absorption coefficient of HbA1c (at 545 nm and 579 nm for level 1, at 544 nm and 577 nm for level 2) are in close agreement with those reported in previous studies. The molar absorption coefficient values were also found to be close to those of earlier reports. The average molar absorption coefficient values of HbA1c were found to be $804,403.5{\;M}^{-1}{\mathrm{cm}}^{-1}$ at 545 nm and $703,704.5\;M^{-1}{\mathrm{cm}}^{-1}$ at 579 nm for level 1 as well as 503,352.4 $M^{-1}{\mathrm{cm}}^{-1}$ at 544 nm and 476,344.6 $M^{-1}{\mathrm{cm}}^{-1}$ at 577 nm for level 2. Our experiments focused on calculating the molar absorption coefficients of HbA1c in the visible wavelength region, and the proposed experimental method has an advantage of being able to easily obtain the molar absorption coefficient at any wavelength in the visible wavelength region. The results of this study are expected to help future investigations on noninvasive methods of estimating HbA1c levels.

## 1. Introduction

Diabetes is a serious lifestyle disease affecting the production and utilization of insulin in the human body and results from the abnormal behavior of glucose consumption by the cells in the body; it can also cause other serious diseases apart from the insulin disorder. Therefore, monitoring and maintaining blood glucose levels by diagnosing diabetes in the early stage is very important. Of all the tests available for diagnosing diabetes, the level of glycated hemoglobin (HbA1c) in the blood can provide a direct indication of blood sugar levels without significant daily fluctuations. The reason for the HbA1c being free from fluctuations is that the value is obtained from a slow reaction of hemoglobin molecules with sugar, which reduces the high-frequency noise in blood glucose levels. For this reason, glycated hemoglobin is considered a weighted average of blood glucose for about 3 to 4 months. According to National Glycohemoglobin Standardization Program (NGSP), HbA1c levels less than 5.7% are considered normal. Values in the range of 5.7% to 6.4% are considered indicative of prediabetes, and values above 6.4% are considered indicative of type 2 diabetes [1].

Many blood glucose sensors are being studied by researchers over the past several years [2,3,4,5,6]. However, most of the sensors are used in an invasive manner and are targeted at improving the estimation accuracy. Some of the implementations are targeting noninvasive approaches, which can reduce the burden of taking a blood sample using a lancet. The invasive HbA1c measurement processes include four major processes [7], and among them, high-performance liquid chromatography (HPLC) and its derivatives are widely adopted. Another invasive process uses several glycation biomarkers apart from HbA1c for diagnosing diabetes [8]. 

On the other hand, noninvasive glucose measurement has been studied for a long time. Different bodily fluids were also used to estimate the blood glucose levels [9,10]. Another form of noninvasive technique is the optical estimation of blood glucose levels [11]. Although optical estimation of glucose level is still insufficient to produce reliable results [12], optical processes for estimating blood glucose levels are of emerging interest [10,13,14,15,16,17]. Among them, [13] focused only on the sensor design, and [14] could not prove to be a viable alternative solution to the state-of-the-art invasive method of blood glucose measurement. In our previous study, we proposed the photoplethysmography (PPG)-based noninvasive estimation method of HbA1c levels using a Beer–Lambert-based model [15]. The estimation of glycated hemoglobin was limited to the process of a simple model considering only absorption. We also developed a finger model utilizing photon diffusion theory and solved a model for estimating the level of glycated hemoglobin in blood using a genetic symbolic regression method [16]. These models have been implemented as transmissive and reflective systems. In [17], a noninvasive method to estimate HbA1c was proposed using machine learning. After extracting the discriminative and effective features from the PPG signal obtained using the developed device, a machine learning algorithm was applied to estimate HbA1c values from the extracted features.

In medical applications, PPG is a noninvasive optical measurement method [18]. To obtain a PPG waveform, optoelectronic components are required, which contain a light source to illuminate the tissue (e.g., skin) and a photodetector (PD) to measure variations in blood volume through changes by the received light intensity. As the PPG signal is a direct result of the change in the volume of blood, the signal can be utilized to measure individual components of a blood solution containing HbA1c compounds. PPG signals are optical signals, so reverse calculation of the optical interactions in a solution can be used to estimate the HbA1c level in practical scenarios. Estimation of the molar absorption coefficient of HbA1c enables calculation of the optical interactions and, hence, the HbA1c level. In [19], the authors presented an experimental setup to measure the molar absorption coefficients of Hb and HbO at near-infrared (NIR) wavelengths; after obtaining control glycohemoglobin solutions, the molar absorption coefficient was estimated using different sets of light-emitting diodes (LEDs) and PDs at different wavelengths. In [20], absorption spectroscopy was used to determine the percentage values of HbA1c for comparison with values from the standard ion-exchange HPLC technique. The hemolysate blood samples of both diabetic and nondiabetic adults were considered, and an absorbance spectrum of glycated hemoglobin was obtained in the range of 190 nm to 880 nm.

In this study, we have measured the molar absorption coefficient of HbA1c molecules through experimentation. The measurements were performed in the wavelength range of 300 nm to 1100 nm and faithfully reported for the range of 450 nm to 700 nm with a low error margin. Thereafter, the results were compared with previously reported molar absorption coefficients for HbA1c based on theoretical [21] and experimental [13] methods. The main contribution of this study can be summarized as follows. Our experimental results demonstrate the validity of the noninvasive glycated hemoglobin estimation results in our previous studies [15,16], to which the theoretically obtained molar absorption coefficient was applied. Moreover, these results are also expected to help improve the accuracy of noninvasively detected HbA1c levels since the proposed experimental method makes it easy to obtain the molar absorption coefficient at any wavelength within a given wavelength range.

## 2. Methodology

### 2.1. Theoretical Method

The molar absorption coefficient of HbA1c was theoretically measured in a previous study [21]. In that study, we estimated the molar absorption coefficient of HbA1c from 300 nm to 1100 nm using a percent transmission data of HbA1c dissolved in water and two references for the molar absorption coefficient of HbA1c at 535 nm and 593 nm [13]. Beer–Lambert law [22] indicates a linear relationship between the absorbance and concentration of an absorbing species. The general Beer–Lambert law can be written as Equation (1), where *A* is the measured absorbance, N is the number of attenuating species, ϵ is the molar absorption coefficient, *c* is the concentration of the compound in solution, and d is the optical path length.

(1) $$A=\overset{N}{\underset{i=1}{\sum }}A_{i}=\overset{N}{\underset{i=1}{\sum }}\epsilon_{i}\times c_{i}\times d=-\log\left(\frac{I}{I_{0}}\right)$$

**Figure 1:**
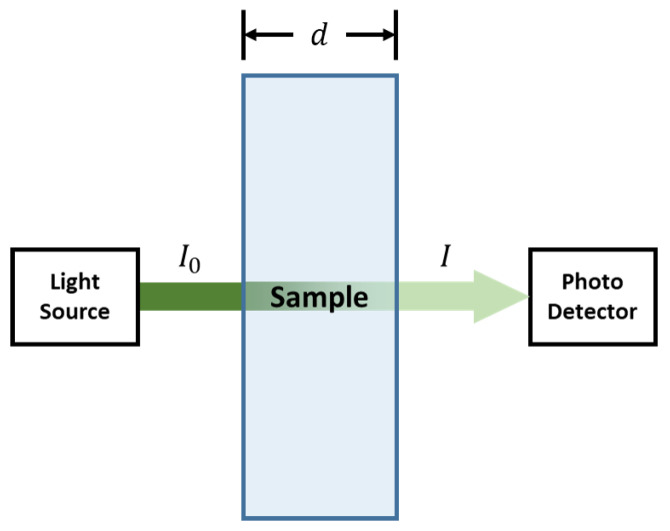
Illustration of Beer–Lambert law [15].

Many compounds absorb ultraviolet (UV) or visible light. Figure 1 shows a beam of monochromatic radiation of radiant intensity *I*_0_ directed at a sample solution. Absorption occurs and the beam of radiation leaving the sample has a radiant intensity *I*.

The amount of radiation absorbed may be measured in several ways:

(2) $$\mathrm{Transmittance},\;T=I/I_{0}$$

(3) $$\%\;\mathrm{Transmittance}\;\left(\%T\right)=100\;T$$

(4) $$\mathrm{Absorbance},\;A=-\log T=\log\left(100/\%T\right)=2-\log\left(\%T\right)$$

From Equation (4), we can easily calculate the absorbance from the percentage transmittance data. The control solutions used contain water as the solvent. From the percent transmittances of water and HbA1c solution for the same optical path length, the absorption of each part can be calculated using Equation (4) to obtain Equations (5) and (6), respectively.

(5) $$A_{HbA1c\_sol}=2-\log\left(\%T_{HbA1c\_sol}\right)$$

(6) $$A_{w}=2-\log\left(\%T_{w}\right)$$

Then, according to Beer–Lambert law from Equation (1), the absorption coefficient is

(7) $$C_{A}=\epsilon c=\frac{A}{d}$$

Now, we can write the full equation of absorption of the solution as

(8) $$A_{HbA1c\_sol}=A_{w}+A_{HbA1c}$$

This equation can be rewritten for the absorption of HbA1c as

(9) $$A_{HbA1c}=A_{HbA1c\_sol}-A_{w}$$

Calculating absorption of a material in this manner results in scaling errors if the transmittance values are normalized. Thus, we denote the actual value of absorption of HbA1c as $A_{HbA1c}^{R}$ and we can let

(10) $$A_{HbA1c}^{R}=k\times A_{HbA1c}$$

If the transmittance values are not normalized, then the value of *k* is taken as 1; for any other normalization factor, the coefficient would have a nonunit value. For both cases (normalized or actual percent transmittance values), the following equation is satisfied. From Equation (1), we can replace $A_{HbA1c}$ in Equation (10) as

(11) $$A_{HbA1c}^{R}=k\times \epsilon_{HbA1c}\left(\lambda \right)\times c_{HbA1c}\times d$$

For the same solution used in the experiment, the molar concentration $c_{HbA1c}$ and optical path d remain the same. Hence, we consider these two parameters in Equation (11) as constants to obtain

(12) $$k^{\prime }=k\times c_{HbA1c}\times d$$

(13) $$A_{HbA1c}^{R}=k^{\prime }\times \epsilon_{HbA1c}\left(\lambda \right)$$

Now, we can state that any value for the absorption of HbA1c at a certain wavelength is directly proportional to the molar absorption coefficient of HbA1c at that wavelength, as shown in Equation (14). Then, Equation (14) can be rewritten for two different wavelengths as shown in Equation (15):

(14) $$A_{HbA1c}^{R}\propto \epsilon_{HbA1c}\left(\lambda \right)$$

(15) $$∴\;\frac{A_{HbA1c}^{R}\left(1\right)}{A_{HbA1c}^{R}\left(2\right)}=\frac{\epsilon_{HbA1c}\left(\lambda_{1}\right)}{\epsilon_{HbA1c}\left(\lambda_{2}\right)}$$

Now, if a reference molar absorption coefficient value is known for any wavelength, then the molar absorption coefficient for the full spectrum can be calculated by the following equation:

(16) $$\epsilon_{HbA1c}\left(\lambda_{2}\right)=\frac{A_{HbA1c}^{R}\left(2\right)}{A_{HbA1c}^{R}\left(1\right)}\times \epsilon_{HbA1c}\left(\lambda_{1}\right)$$

**Figure 2:**
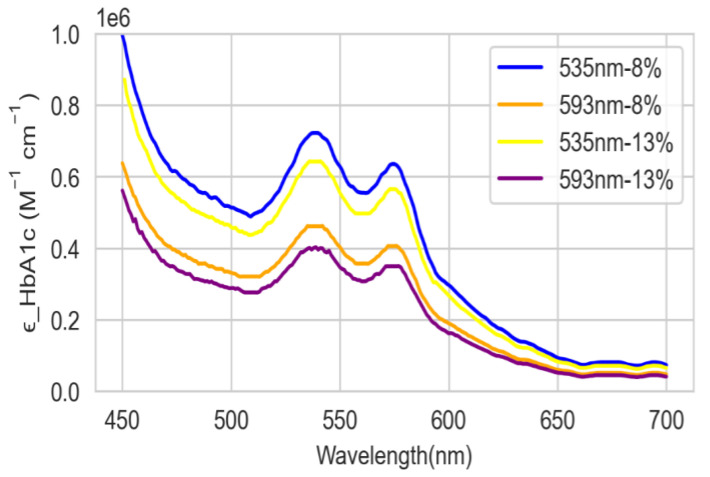
Theoretically calculated molar absorption coefficients of HbA1c [21].

The estimated molar absorption coefficients in the range of 450 nm to 700 nm are depicted in Figure 2 [21]. Note that it was redrawn at the same scale, in the same wavelength range, in order to compare with the experimental results.

### 2.2. Proposed Experimental Method

The absorption coefficients of HbA1c are estimated using a spectrometer (Stellar Net BLACK-Comet-SR Spectrometer), a variable fiber-optical attenuator for multimode patch cables (Thorlabs, Newton, NJ, USA), and polystyrene cuvettes of 1 cm length. The attenuator was used to reduce the intensity of the 5 W tungsten halogen light source. The spectrometer provides a spectrum ranging from 300 nm to 1100 nm and measures the absorbances of different solutions used in the experiment.

**Figure 3:**
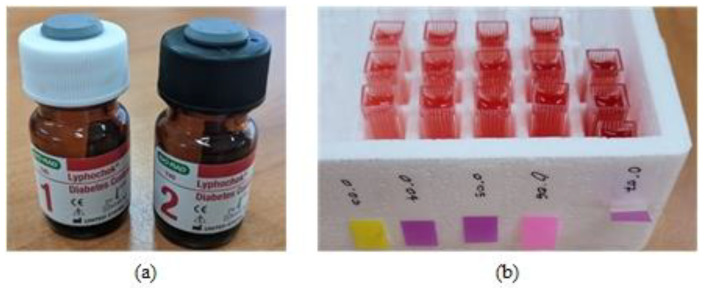
Sample preparation: (**a**) Bio-Rad Lyphocheck diabetes management bilevel solution; (**b**) sample solutions of different concentrations for levels 1 and 2.

A commercially available chemical marker, Bio-Rad Lyphocheck diabetes control bilevel solution (LOT-85821, 85822, Bio-Rad Laboratories, Hercules, CA, USA), was used to prepare the glycated hemoglobin solutions. This marker is a lyophilized whole-blood-based control used to track the clinical assay precision of hemoglobin fractions associated with diabetes, such as hemoglobin A1, A1c, F, total glycated hemoglobin, and total hemoglobin [23]. The glycated hemoglobin solutions were made from the Bio-Rad Lyphocheck diabetes management bilevel solution (Figure 3a). The two levels (1 and 2) were first reconstituted with 0.5 mL of distilled water, and the solutions were allowed to stand for 5–10 min with occasional gentle swirling. The reconstituted blood samples were then diluted to a total hemoglobin content of 0.07 mmol/L. The samples were again diluted to prepare four concentrations of hemoglobin content: 0.06 mmol/L, 0.05 mmol/L, 0.04 mmol/L, and 0.03 mmol/L. This procedure was followed for both levels, and the sample solutions are shown in Figure 3b. The total hemoglobin concentrations were reported to be 9.43 mmol/L for level 1 and 7.44 mmol/L for level 2. The A1c concentrations in the two solutions were 0.228 mmol/L and 0.488 mmol/L for level 1 and level 2, respectively [24]. The diluted solutions were placed in 1 cm cuvettes for each solution and measured three times.

**Figure 4:**
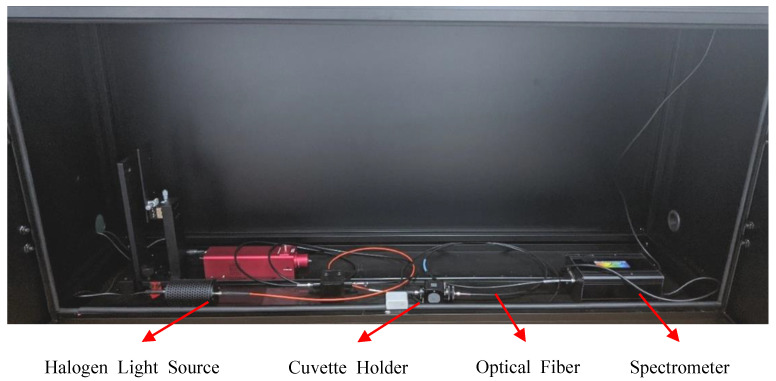
Experimental setup for the measurement of the molar absorption coefficient of HbA1c.

We used a 5 W tungsten halogen light source to produce light in the range of 300 nm to 1100 nm. Figure 4 shows the experimental setup for measuring the molar absorption coefficients of the different solutions. A black-box spectrometer was used and the ambient light in the laboratory was minimized as much as possible. The cuvette holder was stabilized after each sample placement.

**Figure 5:**
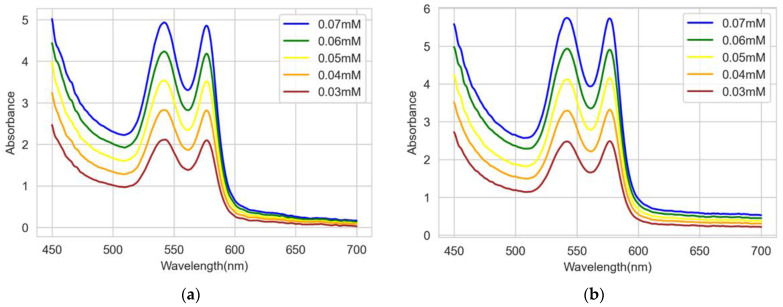
Absorbance spectra of Bio-Rad Lyphocheck diabetes solutions for different concentrations for (**a**) level 1 and (**b**) level 2.

The absorbance was measured for all sample solutions at a total of five concentrations using a spectrometer. Figure 5a shows the absorbance spectra of the level 1 Bio-Rad Lyphocheck diabetes solutions for different concentrations, and Figure 5b shows the absorbance spectra of the level 2 Bio-Rad Lyphocheck diabetes solution for different concentrations.

We measured the molar absorption coefficients of HbA1c through experimentations for comparison with the theoretical values. Note that the molar absorption coefficient of HbA1c can be calculated from the measured absorbance from the spectrometer as follows. In accordance with the Beer–Lambert law [22], as described in Section 2.1, the total absorbance considering the glycated and nonglycated hemoglobin components in blood can be expressed as Equation (17).

(17) $$A_{HbA1c\_sol}=A_{HbA1c}+A_{nonHbA1c\;}$$

The following equation can be written from Equation (17) using Equation (7), where the cuvette length d = 1 cm.

(18) $$\epsilon_{HbA1c\_sol}c_{HbA1c\_sol\;}=\epsilon_{HbA1c}c_{HbA1c\;}+\epsilon_{nonHbA1c}c_{nonHbA1c\;}$$

Then,

(19) $$\epsilon_{HbA1c}=\frac{\epsilon_{HbA1c\_sol}c_{HbA1c\_sol\;}-\epsilon_{nonHbA1c}c_{nonHbA1c}}{c_{HbA1c}}$$

(20) $$=\frac{\epsilon_{HbA1c\_sol}-\epsilon_{nonHbA1c}\frac{c_{nonHbA1c\;}}{c_{HbA1c\_sol\;}}}{\frac{c_{HbA1c\;}}{c_{HbA1c\_sol\;}}}$$

(21) $$=\frac{\epsilon_{HbA1c\_sol}-\epsilon_{nonHbA1c}\left(1-HbA1c\right)}{HbA1c}$$

In Equation (21), the $\epsilon_{\mathrm{nonHbA}1c}$ values are taken from [25].

## 3. Results and Discussion

### 3.1. Experimental Results

**Figure 6:**
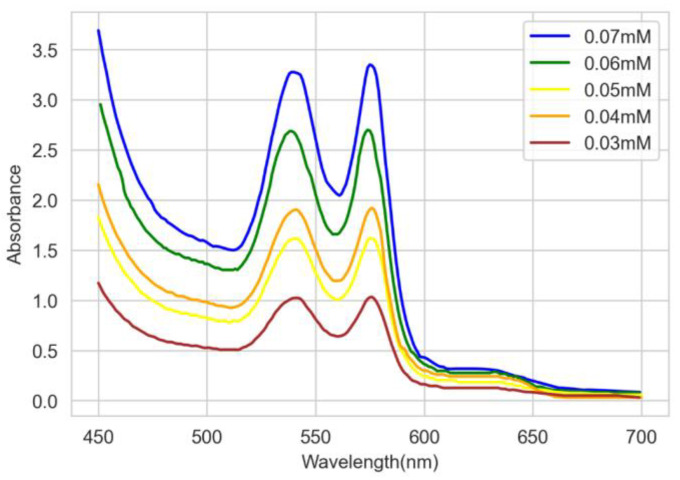
Absorbance spectra from [13].

Figure 5a,b show the absorbance spectra of solutions of different concentrations from 0.03 to 0.07 mM of HbA1c at room temperature for levels 1 and 2, respectively. It is observed that between 500 nm and 600 nm, there are two absorption peaks at around 545 nm and 579 nm. This is well aligned with the measurements from a previous study [13]. To prove this, the absorption spectrum of [13] was redrawn, as shown in Figure 6, which is then used for comparison with our results.

**Figure 7:**
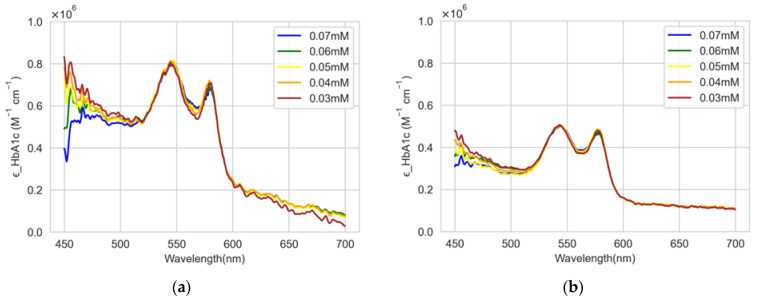
Calculated molar absorption coefficients from the experimental absorbance data. (**a**) Level 1; (**b**) Level 2.

Utilizing the absorbance values from the spectrometer, the molar absorption coefficient of HbA1c in solution, $\epsilon_{HbA1c\;sol}$, can be derived using Equation (1). Subsequently, the actual molar absorption coefficient of HbA1c, $\epsilon_{HbA1c}$, is calculated using Equation (21). The calculated molar absorption coefficients from the experimental absorbance data are plotted in Figure 7a,b for levels 1 and 2, respectively.

**Figure 8:**
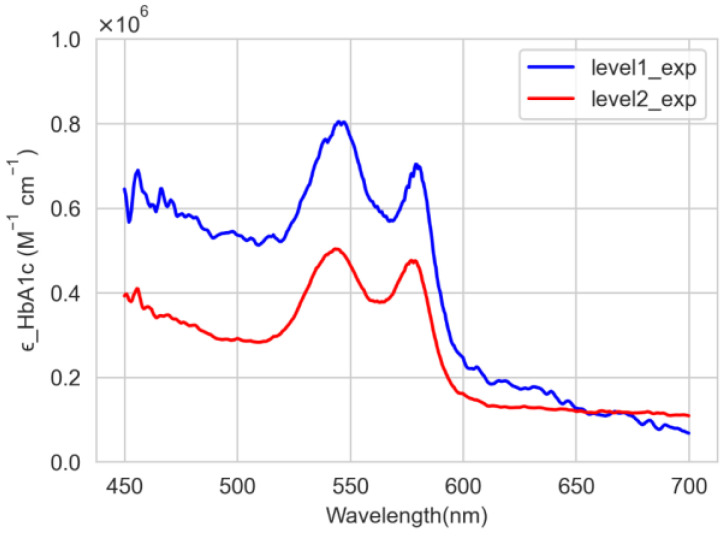
Experimentally calculated mean molar absorption coefficients for levels 1 and 2.

Figure 8 shows the average values of the molar absorption coefficients for levels 1 and 2. At level 1, the peaks are observed at 545 nm and 579 nm, and the corresponding molar absorption coefficients are $804,430.5{\;M}^{-1}{\mathrm{cm}}^{-1}$ and $703,704.5\;M^{-1}{\mathrm{cm}}^{-1}$, respectively. For level 2, the peaks are found at 544 nm and 577 nm, and the corresponding molar absorption coefficients are 503,352.4 $M^{-1}{\mathrm{cm}}^{-1}$ and $476,344.6M^{-1}{\mathrm{cm}}^{-1}$, respectively.

### 3.2. Comparison and Discussion

**Figure 9:**
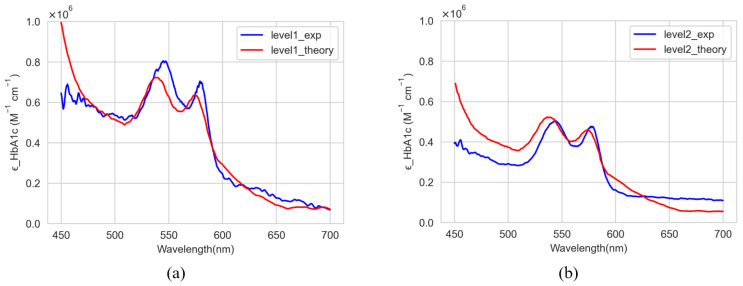
Comparison of the average molar absorption coefficients of HbA1c between the theoretically and experimentally calculated results from 450 nm to 700 nm. (**a**) Level 1; (**b**) Level 2.

Figure 9 shows the comparison between the theoretically (Figure 2) and experimentally (Figure 8) calculated molar absorption coefficients of HbA1c for levels 1 and 2. The numerical results of the comparison are summarized in Table 1.

**Figure 10:**
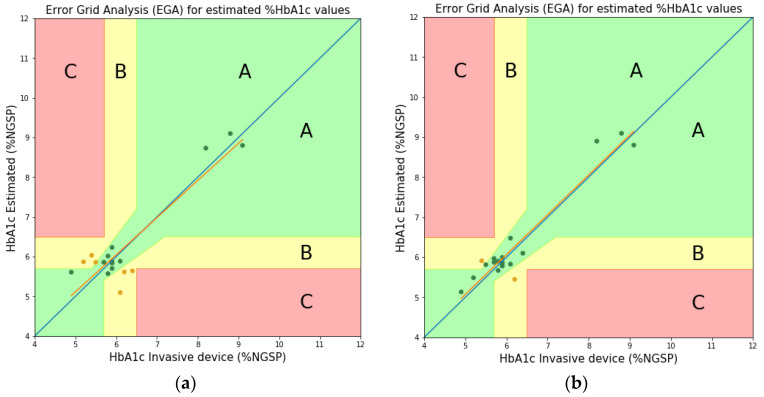
HbA1c Clarke’s error grid analysis (EGA) based on theoretically obtained molar absorption coefficients [15]. (**a**) Blood vessel model; (**b**) Whole finger model.

**Figure 11:**
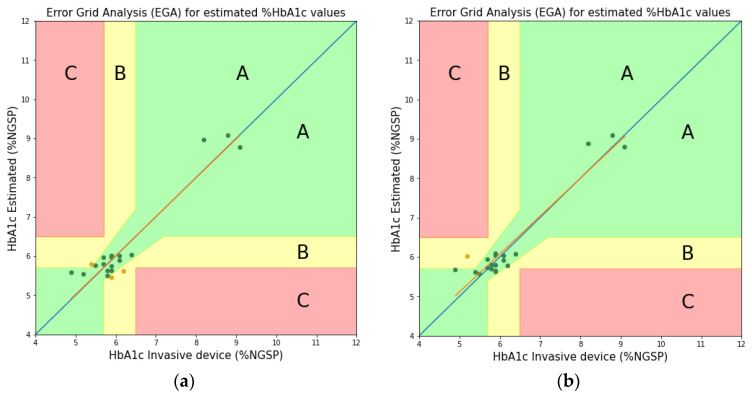
HbA1c Clarke’s error grid analysis (EGA) based on experimentally obtained molar absorption coefficients (Proposed). (**a**) Blood vessel model; (**b**) Whole finger model.

As seen in Figure 9 (or Table 1), between the theoretically and experimentally obtained values, although the peak values are somewhat different, the peak trends are similar and the peak value difference is not large. In [15], the molar absorption coefficients theoretically obtained at three wavelengths (Red, Green, and Blue) were applied to estimate the HbA1c. In this study, the Clarke’s error grid analysis (EGA) plots [26] were used to compare and analyze the accuracy with the estimate of HbA1c obtained by applying the experimentally obtained molar absorption coefficients at the same three wavelengths. Table 2 shows the values of the molar absorption coefficients obtained theoretically and experimentally (level 2) at three wavelengths. In addition, Figure 10 and Figure 11 show the EGA plots when the theoretically obtained molar absorption coefficients are used and the EGA plots when the experimentally obtained molar absorption coefficients are used, respectively. In Figure 10 and Figure 11, we obtained the three zones of usability of the predictions. Zone A represents the values (green dots) within 20% of the reference sensor. Zone B contains points (yellow dots) that are outside of 20% but would not lead to inappropriate treatment. Zone C are those points (red dots) leading to unnecessary treatment. Blue line represents perfect agreement between the reference and estimated values whereas orange line represents the trend line of estimated data. In Figure 10a, the blood vessel model has 14 samples (73.68%), 5 samples (26.31%), and 0 samples (0%) in zones A, B, and C, respectively. Furthermore, in Figure 10b, the whole finger model has 18 samples (90.0%), 2 samples (10.0%), and 0 samples (0%) in zones A, B, and C, respectively [15]. In contrast, Figure 11a illustrates the error grid analysis of the blood vessel model, with zone A containing 16 samples (84.21%), zone B containing 3 samples (15.79%), and zone C with 0 (0%). Figure 11b shows the whole finger model consisting of 19 (95.0%), 1 (5.0%), and 0 (0%) samples in zones A to C, respectively. Therefore, although EGA plots in Figure 10 look almost similar to those in Figure 11, we can say that the accuracy of the HbA1c estimate based on the experimentally obtained molar absorption coefficient is slightly better than that based on the theoretically obtained molar absorption coefficient. Through this comparative analysis, we expect to be able to not only prove the validity of the HbA1c value estimated by applying the theoretically obtained molar absorption coefficient, but also to estimate the HbA1c value more reliably and accurately by applying the experimentally obtained molar absorption coefficient.

In [13], the molar absorption coefficients were obtained only at 535 nm and 593 nm, so we have also calculated the molar absorption coefficient values at the same wavelengths for comparison, in the proposed experimental method, as shown in Table 3. We can see that the molar absorption coefficients obtained by the proposed method are very close to those obtained by the method in [13]. Furthermore, note that the theoretically calculated molar absorption coefficients [21] were obtained based on the reference values at wavelengths 535 nm and 593 nm in [13].

Our experiments focused on calculating the molar absorption coefficients of HbA1c in the visible wavelength region, and the proposed experimental method has a greater advantage, of being able to easily obtain the molar absorption coefficient at any wavelength in the visible wavelength region, than that of [13], and may ultimately facilitate further analysis of noninvasive HbA1c estimation. It is important to note that, in our previous studies [15,16], we applied the theoretically obtained molar absorption coefficients at three wavelengths of R (625 nm), G (525 nm), and B (465 nm) of a white LED to estimate HbA1c noninvasively. In this study, we considered the region from 450 nm to 700 nm, and there is minimal noise in the experimental data obtained in this range. However, some internal noises from the spectrometer can be found near 450 nm. The measurements were performed carefully to maintain homogeneity and sensitivity. Moreover, some other probable sources of error can be environmental influences, instrument fluctuations, and line-voltage variations. One of the limitations of this study is that the range must be reduced across the entire spectrum to reduce the margin of error. Our future work will involve obtaining absorbance and corresponding molar absorption coefficient values of HbA1c over the full spectrum from 300 nm to 1100 nm with a relatively low margin of error. Then, we expect that the accuracy and reliability of the noninvasive HbA1c estimates can also be improved.

## 4. Conclusions

In this study, we focused on the experimental calculation of the molar absorption coefficients of glycated hemoglobin, HbA1c, from 450 nm to 700 nm using an optical method. The experimental calculations of the molar absorption coefficients of HbA1c were performed and compared with data from other studies. According to our findings, we observed two distinct peaks at 545 nm and 579 nm for level 1 and at 544 nm and 577 nm for level 2, respectively. The average values of the molar absorption coefficients of HbA1c were found to be $804,430.5{\;M}^{-1}{\mathrm{cm}}^{-1}$ at 545 nm and $703,704.5{\;M}^{-1}{\mathrm{cm}}^{-1}$ at 579 nm for level 1 as well as 503,352.4 $M^{-1}{\mathrm{cm}}^{-1}$ at 544 nm and 476,344.6 $M^{-1}{\mathrm{cm}}^{-1}$ at 577 nm for level 2. Compared to theoretically calculated values, we have shown that the peak trend is similar and that the peak value difference is not large. Furthermore, we experimentally calculated the molar absorption coefficients at 535 nm and 579 nm for comparisons with the results of [13] and showed that the molar absorption coefficients obtained by the proposed experimental method are very close to those reported in [13]. Our proposed experimental method thus makes it easy to obtain the molar absorption coefficient at any wavelength within a given wavelength range, and applying this method is expected to improve the accuracy and reliability of noninvasive HbA1c estimates.

## Figures and Tables

**Table 1:** Comparison of the molar absorption coefficients of HbA1c at two peak wavelengths for the theoretically and experimentally obtained results.

Wavelengths at Two Peaks		$\mathrm{Level}\;1\;[M^{-1}cm^{-1}]$	$\mathrm{Level}\;2\;[M^{-1}cm^{-1}]$
Theoretical [21]	540 nm	722,205.0	520,757.0
	576 nm	632,437.9	455,116.5
Experimental (Proposed)	545 (544) * nm	804,430.5	503.352.4
	579 (577) * nm	703,704.5	476,344.6

( ) *: Wavelength corresponding to the peak at level 2.

**Table 2:** Molar absorption coefficients of HbA1c obtained theoretically and experimentally at level 2.

Wavelength (nm)	Molar Absorption Coefficients of HbA1c (M^−1^ cm^−1^)	
	Theoretically [21]	Experimentally (Proposed)
465	549,025	342,907
525	455,140	341,034
615	170,555	131,187

**Table 3:** Comparison of the measured molar absorption coefficients of HbA1c between the present study and Reference [13].

Wavelength		Level 1	Level 2
Experimental (Proposed)	535 nm	710,223.1	457,408.7
	593 nm	322,781.2	203,323.9
Reference [13]	535 nm	710,888	456,061
	593 nm	322,197	201,765

## Data Availability

The dataset used in this research is available upon a valid request to any of the authors of this research paper.
